# Neural Correlates of Dual-Task Walking: Effects of Cognitive versus Motor Interference in Young Adults

**DOI:** 10.1155/2016/8032180

**Published:** 2016-04-20

**Authors:** Rainer Beurskens, Fabian Steinberg, Franziska Antoniewicz, Wanja Wolff, Urs Granacher

**Affiliations:** ^1^Research Focus Cognition Sciences, Division of Training and Movement Sciences, University of Potsdam, 14469 Potsdam, Germany; ^2^Geriatric Center at the University of Heidelberg, Agaplesion Bethanien Hospital, 69126 Heidelberg, Germany; ^3^Department of Sports Psychology, Institute of Sports Science, University of Mainz, 55122 Mainz, Germany; ^4^Division of Sport and Exercise Psychology, University of Potsdam, 14469 Potsdam, Germany; ^5^Department of Sports Science, Sport Psychology, University of Konstanz, 78464 Konstanz, Germany

## Abstract

Walking while concurrently performing cognitive and/or motor interference tasks is the norm rather than the exception during everyday life and there is evidence from behavioral studies that it negatively affects human locomotion. However, there is hardly any information available regarding the underlying neural correlates of single- and dual-task walking. We had 12 young adults (23.8 ± 2.8 years) walk while concurrently performing a cognitive interference (CI) or a motor interference (MI) task. Simultaneously, neural activation in frontal, central, and parietal brain areas was registered using a mobile EEG system. Results showed that the MI task but not the CI task affected walking performance in terms of significantly decreased gait velocity and stride length and significantly increased stride time and tempo-spatial variability. Average activity in alpha and beta frequencies was significantly modulated during both CI and MI walking conditions in frontal and central brain regions, indicating an increased cognitive load during dual-task walking. Our results suggest that impaired motor performance during dual-task walking is mirrored in neural activation patterns of the brain. This finding is in line with established cognitive theories arguing that dual-task situations overstrain cognitive capabilities resulting in motor performance decrements.

## 1. Introduction

Multitasking during walking is the norm rather than an exception during everyday life. Walking while talking on the cell-phone or while navigating around obstacles represent examples for the concurrent performance of a motor task (i.e., walking) and a cognitive interference task (e.g., talking). There is a plethora of literature indicating that the gait pattern is negatively affected during dual-task walking in young adults [1–4]. In contrast to earlier theories [5], this indicates that supraspinal centers are involved in the coordinated regulation of gait in humans (cf. [6] for a review).

On a behavioral level, Beauchet et al. [4] were able to show that counting backwards while walking provoked a decrease in stride velocity and an increase in stride time variability in healthy young adults. Recently published studies are in line with this finding and revealed significant decreases in young adults' gait speed while performing auditory [3] or visual [1] discrimination tasks. The decrease in walking performance has been attributed to limited cognitive processing capabilities when two or more tasks share the same brain networks [7]. A common theory to explain dual-task interference is the central capacity-sharing model [8]. That is, two or more concurrent tasks interfere when they share the same cognitive resources. In other words, when the primary task demands motor control and the secondary task requires cognitive processing, a decrement in performance of one or both tasks can be observed.

So far, the dual-task paradigm has primarily been examined using behavioral approaches. Researchers investigated whether performance decrements occur in the primary and/or secondary task while walking in dual-task situations. However, there is a gap in the literature regarding the neural correlates of performance changes during single- and dual-task walking. In fact, only few studies examined neural activation during dual-task walking in young adults [9–11]. All found evidence that neural activation in frontal brain regions (measured by functional near-infrared spectroscopy (fNIRS)) increased when young subjects walked while performing a serial subtraction [9] or a verbal fluency task [11] compared to single-task walking. However, the aforementioned studies used fNIRS, a technique that is restricted by a limited number of channels and poor temporal resolution. Thus, only predefined brain areas (i.e., prefrontal brain networks) can be registered.

To date, recent advances in technology (i.e., shielded Ag/AgCl electrodes and artifact removal algorithms [12]) allow the use of electroencephalographic (EEG) systems while walking. EEG systems are better suited to register neural activation across the human scalp with higher temporal resolution. For example, changes in the power of alpha and beta frequency bands during walking have been reported in frontal, central, and parietal areas of the brain in healthy young adults [13]. These frequency bands describe sensory processing and memory (alpha band) [14] as well as attentional and motor processes (beta band) [15, 16]. One of the few studies utilizing EEG during dual-task walking showed that the simultaneous performance of a cognitive interference task (Go/NoGo task) while walking on a treadmill increased cognitive load and altered processing mechanisms in central and frontal brain regions (i.e., FC_z_ and C_z_ electrodes) compared to performing the cognitive task in a seated position [17]. Further, there is preliminary evidence indicating that gamma frequencies were affected in young adults when walking while concurrently performing a motor task (i.e., carrying a glass of water) or an arithmetic task (i.e., serial subtraction of numbers) [13]. The authors reported that gamma oscillations in frontal brain regions increased during the arithmetic interference task but decreased while performing the motor task. Hence, findings on neural correlates of dual-task walking are scarce and ambiguous but might provide valuable insights into single- as compared to dual-task brain activation patterns that go beyond behavioral measures. Further, the serial subtraction task used in the latter study by Marcar et al. [13] required a verbal response, which is known to affect EEG registration by increasing “activation” through face muscle activation rather than the secondary task [18].

Recent findings indicate that the specific demands of a secondary task (i.e., cognitive versus motor interference) diversely affect motor performance during dual-task walking (cf. [2] for a review). Thus, it is imperative and timely to examine whether this is reflected by different neuroelectric brain activation patterns. To our knowledge, there is no study available that investigated the effects of various interference tasks on neural activation during dual-task walking. Further, dual-task walking does not only affect frontal networks but it may additionally impact central and parietal brain areas, depending on task demands [19]. Thus, the aims of this study were to assess neural activation in frontal, central, and parietal brain regions and behavioral performance during single- and dual-task walking in young adults. In addition, applying a cognitive and a motor interference task examined specific effects of the secondary task demands during dual-task walking. We hypothesized that (i) gait characteristics are impaired during dual- as compared to single-task walking, (ii) impairments are modulated by the type of the secondary task with larger performance decrements caused by the motor interference compared to the cognitive interference task, and (iii) these behavioral impairments are reflected in neural activity patterns.

## 2. Methods

### 2.1. Ethics Statement

The Human Ethics Committee at the University of Potsdam approved the study protocol (reference number: 20/2015). Before the start of the study, each participant read, concurred, and signed a written informed consent. All procedures were conducted according to the Declaration of Helsinki.

### 2.2. Participants

A sample of 12 healthy adults (6 males/6 females, age: 20–28 years) participated in the experiments. Their characteristics are summarized in Table 1. None of them had any known neuromuscular or orthopaedic diseases or injuries that may have affected their ability to conduct the experiments. All participants were naïve with regard to research on motor control and cognitive functioning. An a priori power analysis with an actual power of 0.8 using a repeated-measure ANOVA design (one group, three experimental conditions) yielded a total sample size of *N* = 12 (*α* = 0.05; critical *F* = 3.44). Effect size was estimated using previously published work on the effects of attentional-demanding tasks on walking performance (i.e., stride time) in young adults. A delta of 150 ms between single-task (ST) and dual-task (DT) stride time was used to calculate the effect size for the a priori power analysis and resulted in an *f*-value of 0.5 [17].

### 2.3. Experimental Procedure

Prior to the experiment, body height was assessed using a wall-mounted stadiometer (Seca, Basel, Switzerland). In addition, body mass and body composition (i.e., skeletal muscle mass and body fat) were registered by means of a bioimpedance analysis system (InBody 720, BioSpace, Seoul, Korea). Standardized verbal instructions regarding the test procedure were given prior to the experiments. Participants walked back and forth on a 10 m straight path for 2 minutes. Each walk was initiated and terminated one meter before and after the walkway to allow sufficient distance to accelerate and decelerate from a steady-state of ambulation. The experiment was subdivided into three experimental conditions. During single-task walking (ST walk), participants walked at their self-selected walking speed. In the cognitive interference condition (DT-CI), participants conducted the walking task while concurrently performing an attention-demanding interference task. The CI task comprised the random presentation of high-pitched (2,000 Hz) and low-pitched (300 Hz) tones. Subjects were asked to press a button as soon as a low-pitched tone was played and ignore the stimulus when a high-pitched tone was presented (i.e., Go/NoGo task). In the motor interference condition (DT-MI), participants held two sticks, one in each hand, in front of their body. Each stick had a ring at the end (diameter: 4 cm) and the rings were interlocked [20]. A small voltage on the rings enabled the registration of ring contacts. Participants were advised not to let the rings touch each other. In addition, both the CI tasks and the MI task were performed in single-task condition (i.e., while seated, ST-CI and ST-MI) and all experimental conditions were performed in a counterbalanced order.

### 2.4. Assessment of Gait Performance

Gait performance was registered using a 10 m instrumented walkway equipped with an OptoGait-System (Microgate, Bolzano, Italy) [21]. The OptoGait-System is an optoelectrical measurement system consisting of light-transmitting and light-receiving bars. Each bar is one meter in length and consists of 100 LEDs transmitting to an oppositely positioned bar. The continuous connection between two bars allowed measuring and timing of any break in the connection. Spatial and temporal gait characteristics were registered at 1,000 Hz. The OptoGait-System demonstrated high discriminant and concurrent validity with a validated electronic walkway (GAITRite®-System) for the assessment of gait parameters in healthy young subjects [22].* Gait velocity* was defined as distance in meters covered per second during one stride,* stride length* as linear distance in centimeters between two successive heel contacts of the same foot, and* stride time* as time in seconds between the first contacts of two consecutive footfalls of the same foot. In addition, coefficients of variation (CV) for each gait measure were calculated according to the formula [23]:

(1) $$\begin{array}{c} CV\left[\;\%\;\right]=\left(\;\frac{SD}{Mean}\;\right)\times 100. \end{array}$$

### 2.5. Assessment of Secondary Task Performance

Performance in the CI task was assessed by measuring the time between stimulus onset (presenting a low-pitched tone) and the appropriate motor response (pressing a button box). Lower reaction times indicate a better performance. Performance in the MI task was evaluated as the total time of contact between the two interconnected rings. A shorter total contact time between the rings is indicative of better performance [20]. During walking trials (DT-CI and DT-MI), registered data of the cognitive and motor interference tasks recorded at the turning points of a walk were excluded from further analyses.

### 2.6. Assessment of Neural Correlates

A mobile 64-channel EEG system (Advanced Neuro Technology, Enschede, Netherlands) was used to register neural correlates during ST and DT conditions [24]. Electrode position was set according to the International 10-20 standard system [25] with the vertex (electrode: C_z_) positioned halfway between the nasion and inion. Channel data were referenced using the average of all connected electrodes (common average). During EEG recordings, participants were instructed to limit any blinking, jaw clenching, or facial expressions that could introduce artifacts into the EEG signal. EEG data were registered at 1,024 Hz and analyzed offline using the Brain Vision Analyzer (Brain Products, Munich, Germany). The EEG signal was bandpass-filtered (0.5 Hz low-cutoff, 45 Hz high-cutoff filter; time constant: 0.32 s; slope: 48 dB/octave) and corrected for artifacts induced by eye movements [26]. Following a first careful visual inspection and systematic exclusion criteria, the allowance of semiautomatic artifact rejection was set (gradient: <35 mV; amplitude range: −100 to 100 mV) [27]. Subsequently, data were segmented in 1 s segments, analyzed using spectral analysis (FFT) with a resolution of 0.5 Hz, and averaged across a 2-minute trial for each walking condition. Average voltage activity was exported for the following frequencies: alpha band (8–12 Hz) and beta band (13–30 Hz) [14, 28].

### 2.7. Statistical Analyses

Data are presented as means and standard deviations. Gait parameters were analyzed in separate analyses of variance (ANOVA) using the within-factor condition (ST walk, DT-CI, and DT-MI) to describe the effects of the CI and MI task on walking performance. CI and MI task performance was analyzed across conditions using separate one-way repeated-measure ANOVA (within-factor condition: ST-CI/DT-CI; ST-MI/DT-MI). Lastly, neural activation during walking was analyzed at the cranial midline (electrodes: FP_z_, F_z_, FC_z_, C_z_, P_z_, and PO_z_) using separate analyses of variance (ANOVA) with the within-factor attention (ST walk, DT-CI, and DT-MI). Bonferroni-corrected post hoc tests were conducted to identify comparisons that were statistically significant. Effect sizes were determined by calculating Cohen's *d* [29], a measure that defines whether a difference is of practical concern. Cohen's *d* values are classified as follows: 0.00 ≤ *d* ≤ 0.49 indicate small, 0.50 ≤ *d* ≤ 0.79 indicate medium, and *d* ≥ 0.8 indicate large effects [29]. All analyses were calculated using Statistical Package for Social Sciences (SPSS) version 23.0 (IBM Corp., New York, USA) and significance levels were set at *α* = 5%.

## 3. Results

### 3.1. Gait Performance

**Figure 1:**
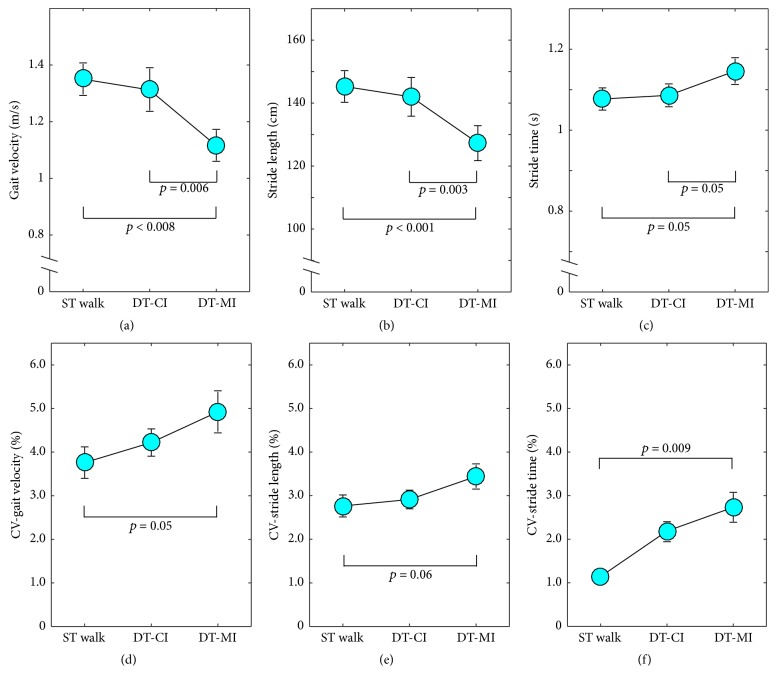
Subjects' mean gait performance and the respective variability measures, separated by condition (ST walk: single-task walking, DT-CI: walking + cognitive interference, DT-MI: walking + motor interference) for (a) gait velocity, (b) stride length, (c) stride time, (d) CV-gait velocity, (e) CV-stride length, and (f) CV-stride time. Circles represent mean values and error bars the respective 95% confidence interval.

On average, subjects walked 136.7 ± 17.2 m during ST walk, 129.2 ± 14.4 m during DT-CI, and 111.7 ± 12.7 m during DT-MI. Figures 1(a)–1(c) display mean gait values and Figures 1(d)–1(f) the respective gait variability. ANOVA yielded a significant main effect of condition for each evaluated gait parameter, namely, gait velocity (*p* < 0.001, *d* = 2.2), stride length (*p* < 0.001, *d* = 2.5), stride time (*p* < 0.01, *d* = 1.5), the variability of gait velocity (*p* < 0.05, *d* = 1.2), stride length (*p* < 0.05, *d* = 1.3), and stride time (*p* < 0.01, *d* = 1.5). Bonferroni-adjusted post hoc tests indicated that gait velocity and stride length decreased and stride time increased during DT-MI compared to ST walking (*p* < 0.01, *d* = 2.0, *p* < 0.001, *d* = 3.0, and *p* < 0.05, *d* = 1.6, resp.) and DT-CI (*p* < 0.01, *d* = 2.2, *p* < 0.001, *d* = 2.4, and *p* < 0.05, *d* = 1.4, resp.). Also, variability in gait velocity, stride length, and stride time significantly increased from ST walking to DT-MI (all *p* < 0.05, *d* = 1.6–2.3).

### 3.2. Secondary Task Performance

ANOVA yielded a significant main effect of condition for MI task performance (i.e., contact time of rings: *p* < 0.01, *d* = 3.3) and CI task performance (reaction time: *p* < 0.01, *d* = 2.3). Reaction time (ST: 282.3 ms, DT: 343.5 ms, Δ61.2 ms) and handle contact time (ST: 19.9 ms, DT: 422.2 ms, Δ402.3 ms) significantly increased during DT compared to ST.

### 3.3. Neural Activation

**Figure 2:**
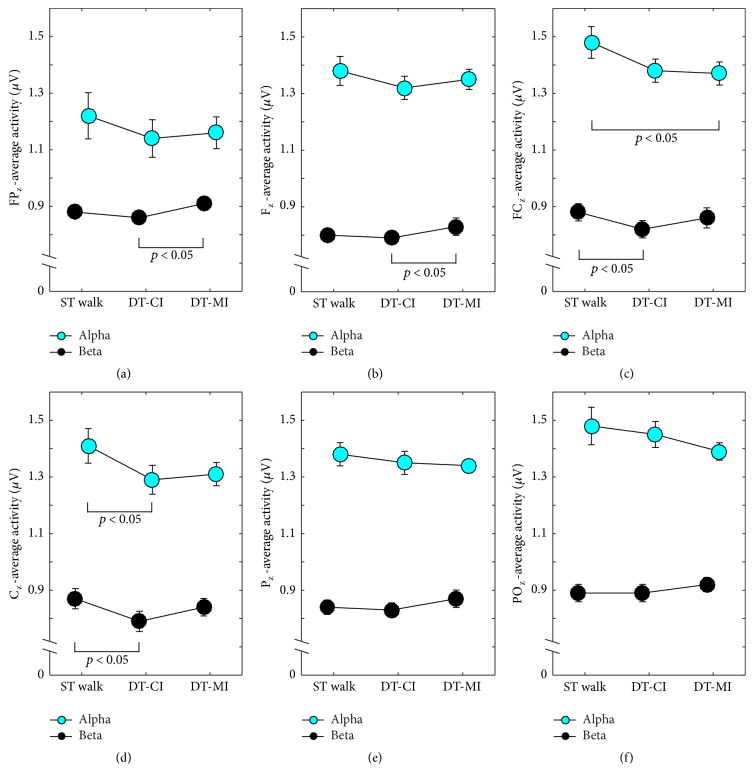
Subjects' mean average voltage across the cranial midline, separated by condition (ST walk: single-task walking, DT-CI: walking + cognitive interference, DT-MI: walking + motor interference). Values represent average voltage for (a) FP_z_, (b) F_z_, (c) FC_z_, (d) C_z_, (e) P_z_, and (f) PO_z_. Cyan circles represent mean alpha frequency; black circles show mean beta frequency; error bars represent the respective 95% confidence interval.

Figures 2(a)–2(f) display average activities across electrodes (FP_z_, F_z_, FC_z_, C_z_, P_z_, and PO_z_) and frequencies (alpha band and beta band). ANOVA outcomes are shown in Table 2. For alpha frequencies, ANOVA yielded a significant main effect of condition for two out of six electrodes, namely, FC_z_ (*p* < 0.05, *d* = 1.2) and C_z_ (*p* < 0.05, *d* = 1.2). Bonferroni-adjusted post hoc tests indicated that average activity significantly decreased during DT-MI in FC_z_ (*p* < 0.05, *d* = 1.5) and during DT-CI in C_z_ (*p* < 0.05, *d* = 1.4) compared to ST walking.

For beta frequencies, ANOVA yielded a significant main effect of condition for four out of six electrodes, namely, FP_z_ (*p* < 0.05, *d* = 1.1), F_z_ (*p* < 0.05, *d* = 1.2), FC_z_ (*p* < 0.05, *d* = 1.1), and C_z_ (*p* < 0.05, *d* = 1.2). Bonferroni-adjusted post hoc tests revealed that average activity in central electrodes (FC_z_, C_z_) significantly decreased during DT-CI (FC_z_: *p* < 0.05, *d* = 2.5; C_z_: *p* < 0.05, *d* = 1.4) compared to ST walking. In frontal electrodes (FP_z_, F_z_), average activity was significantly higher during DT-MI (FP_z_: *p* < 0.05, *d* = 2.0; F_z_: *p* < 0.05, *d* = 1.5) compared to DT-CI.

## 4. Discussion

The present study was designed to examine the role of different secondary task demands (i.e., cognitive and motor interference) on walking performance in young adults and elucidated the associated neural activation patterns. Our main results can be summarized as follows: (1) the MI task but not the CI task affected walking performance in young adults, that is, reduced gait velocity and stride length, increased stride time, and increased tempo-spatial variability, which confirmed our first and second hypothesis; (2) average activity in frontal and central brain regions was modulated during both CI and MI walking conditions, indicating increased cognitive load. Thus, our third hypothesis was also confirmed.

### 4.1. Findings on Walking Performance

Comparable to previous works [3, 4], deficits in DT walking observed in the present study were manifested in reduced walking speed and stride length and increased stride time. Additionally, the tempo-spatial variability of walking (i.e., CV of gait velocity, stride length, and stride time) significantly increased during DT walking, which has been shown previously (cf. [30] for a review). Decreased gait and/or secondary task performance in healthy young adults while walking and concurrently performing attention-demanding tasks can be explained by competing demands for attentional resources involved in both tasks [31], thereby reaching central capacity limits of the brain. In order to compensate for these demands, brain activation is increased [32]. DT-related decreases in walking performance and in both interference tasks (i.e., increased reaction times and contact handle times) found in our study support this argument. However, cognitive interference and motor interference differently affected walking performance. During DT-MI, walking speed and stride length decreased while stride time and tempo-spatial variability increased, which was less pronounced during DT-CI. Explanations for DT interference are based on the assumption that attentional resources are limited [7]. DT interference is likely to occur if the available central capacity is exceeded, which causes an inability to appropriately adapt the allocation of attention between two concurrently performed tasks. The CI task used in our paradigm was relatively easy and might not have been challenging enough to reach the central capacity limit of young adults. Therefore, the attentional load needed to simultaneously perform the CI task and the walking task did not overload the available central resources and thus only provoked little interference with minor gait changes. Walking speed decreased by 2.9%, stride length decreased by 2.3%, and stride time increased by 0.8% during the CI task. Similar results were found previously [4, 33]. For instance, walking while performing an arithmetic [4] or a memorizing task [34] only marginally reduced stride length and gait speed in young adults (5.4% and 3.3%, resp.). On the other hand, the MI task used in our study significantly changed the gait pattern. Walking speed decreased by 15.1%, stride length decreased by 10.4%, and stride time increased by 5.5%. Also tempo-spatial variability increased by 12–20%, which is in line with previous studies using motor interference tasks while walking [35, 36] and showed significant modifications of walking. Grabiner and colleagues [35] found increased stride time variability in young adults while concurrently carrying a cup of water. Similarly, a study by Ebersbach and colleagues [36] reported a significant increase in stride time when young subjects walked and simultaneously performed a finger tapping task. Our findings might be explained by the capacity-sharing model of attention [37]. Two or more concurrent tasks interfere when they share the same cognitive resources. Here, the MI task and the walking task both demand motor control, which is likely to call for the same brain networks. Thus, the interference while performing a motor task while walking might be higher compared to performing a cognitive task and thus performance decrements in both motor tasks occur.

### 4.2. Findings on Neural Correlates

Our results indicate that neural activation in frontal brain regions was modulated while walking in DT situations. This finding is in line with previous studies showing increased prefrontal activation when walking while concurrently performing a serial subtraction [9] or a verbal fluency task [11] in young adults. In our study, lower alpha activity in frontal brain areas was demonstrated when walking while concurrently performing the CI and the MI task, which is indicative of an increased cognitive load during both DT walking conditions [14]. In contrast to previous studies [9–11], our registration of neural activity was not limited to frontal brain regions. We were able to register neural activity throughout the cranial midline (i.e., frontal through parietal cortex). Our findings showed that alpha activity during DT walking was also reduced in central brain regions. The decrease in alpha frequencies during the CI task might be explained by the argument that alpha frequencies are affected by the expectation of an auditory stimulus [38] or the inhibition of responses [15]. Subjects in our experiment were asked to respond to a low-pitched tone by pressing a button and inhibit their response to a high-pitched tone. Thus, cognitive load increased in frontal (processing of the auditory stimulus [38]) and in central regions (processing of the motor response [39]) during DT compared to ST walking. In contrast to the CI task, the MI task primarily demanded motor control (i.e., hold the two interlocked sticks), which caused modulations of the alpha band in frontal areas of the brain [39]. Additionally, subjects needed to share attention between the MI task and the walking task. It appears reasonable to argue that deficits in walking performance occur when subjects are forced to coordinate two different sources of visual information, one related to walking through visually defined space [40] and the other to the performance of the nonwalking MI task. Since the coordination of multiple tasks is an executive function, thought to be located in the prefrontal cortex, the observed increase of cognitive load in frontal brain regions supports this assumption.

In contrast to alpha activity, beta activity in frontal brain regions (FP_z_, F_z_) was increased when performing the MI task compared to performing the CI task during walking. Also, a tendency towards higher activity during DT-MI compared to ST walking was found. This indicates that the type of the secondary task (motor interference versus cognitive interference) modulated brain activation patterns in young adults. In general, beta activity increases when the cognitive load increases [15]. In addition, beta activity increased when performing motor-demanding tasks (i.e., grasping tasks) [16] and is particularly pronounced during isometric compensation of low-level forces [41]. The MI task in our study required holding two interlocked sticks. Thus, our findings support the assumption that the relatively low physical demand of holding the handles was suitable to increase beta activity in our subjects. Also, increased beta activity has been shown when a motor task is disturbed and the former steady-state had to be reestablished (cf. [28] for a review). That is, the demand of the MI task used in our study affected subjects' walking performance and more attentional demand was needed to compensate for this disturbance (i.e., increased beta activity). However, our results also showed that, in more central electrodes (FC_z_, C_z_), beta activity significantly decreased during walking while performing the CI task. Alegre and colleagues [19] reported that beta activity is related to motor preparation and inhibition in a Go/NoGo paradigm. The Go/NoGo task used in our experiments included action trials (i.e., trials where subjects had to respond to low-pitched triggers) and inhibition trials (i.e., trials where subjects had to inhibit responses to high-pitched triggers). Similar to our findings, authors found decreased beta activity in central brain regions during action trials and increased beta activity during inhibition trials. It is argued that decreased beta band activity was associated with motor preparation and execution. However, this decrease in activity was followed by a postmovement increase (i.e., beta rebound), presumably reflecting processes to reset the motor networks [19]. The general decrease in beta activity during DT-CI in our study might be due to our methodological approach. We averaged activity over the complete DT-CI walking trial, thus integrating action trials as well as inhibition trials. It may be possible that the decrease in beta activity during action trials was more pronounced than the increase in inhibition trials and thus a general decrease was observed. Also, previously conducted studies used the Go/NoGo task in ST condition only. We performed the Go/NoGo task while walking. Since activation processes in beta frequencies are primarily associated with motor processes [16, 42], our methodological approach did not allow us to discriminate effects of walking from the specific effects of action/inhibition trials during DT walking.

## 5. Conclusion

In conclusion, our results indicate that impaired motor performance during DT walking is mirrored in neural activation patterns of the brain. Gait performance decreased during cognitive and motor interference while walking. We were also able to show increased cognitive load during both CI and MI walking conditions compared to ST walking. This finding is well in line with established cognitive theories arguing that DT situations overstrain cognitive capabilities [43], resulting in motor performance decrements.

## Figures and Tables

**Table 1:** Participants' characteristics.

	Total (*n* = 12)	Female (*n* = 6)	Male (*n* = 6)
Age [years]	23.8 ± 2.8	24.3 ± 2.8	23.3 ± 3.0
Height [cm]	174.3 ± 12.6	166.0 ± 9.2	182.5 ± 9.9
Mass [kg]	68.0 ± 13.0	58.6 ± 7.6	77.5 ± 10.1
BMI [kg/m^2^]	22.2 ± 1.8	21.1 ± 0.6	23.3 ± 2.0
SMM [kg]	31.8 ± 8.1	25.7 ± 4.2	37.8 ± 6.3
FM [kg]	11.6 ± 1.7	12.2 ± 1.8	11.1 ± 1.6

BMI: body mass index, SMM: skeletal muscle mass, and FM: fat mass.

**Table 2:** ANOVA outcome for average activity [*μ*V] in alpha/beta frequencies (mean ± SD).

(A) Alpha band (electrodes)	ST walk	DT-CI	DT-MI	Condition *p* value (*d*)
FP_z_	1.22 ± 0.3	1.19 ± 0.2	1.18 ± 0.2	0.75 (0.4)
F_z_	1.38 ± 0.2	1.35 ± 0.1	1.35 ± 0.1	0.53 (0.5)
FC_z_	1.48 ± 0.2	1.41 ± 0.1	1.38 ± 0.1	**0.03 (1.2)**
C_z_	1.41 ± 0.2	1.31 ± 0,2	1.32 ± 0.1	**0.04 (1.2)**
P_z_	1.38 ± 0.1	1.35 ± 0.1	1.35 ± 0.1	0.55 (0.5)
PO_z_	1.48 ± 0.2	1.43 ± 0.2	1.39 ± 0.1	0.10 (1.0)

(B) Beta band (electrodes)	ST walk	DT-CI	DT-MI	Condition *p* value (*d*)

FP_z_	0.88 ± 0.1	0.85 ± 0.1	0.90 ± 0.1	**0.04 (1.1)**
F_z_	0.80 ± 0.1	0.80 ± 0.1	0.84 ± 0.1	**0.04 (1.2)**
FC_z_	0.88 ± 0.1	0.84 ± 0.1	0.86 ± 0.1	**0.04 (1.1)**
C_z_	0.87 ± 0.1	0.79 ± 0.1	0.84 ± 0.1	**0.03 (1.2)**
P_z_	0.84 ± 0.1	0.84 ± 0.1	0.87 ± 0.1	0.26 (0.7)
PO_z_	0.89 ± 0.1	0.91 ± 0.1	0.92 ± 0.1	0.21 (0.8)

ST walk: single-task walking, DT-CI: walking + cognitive interference, DT-MI: walking + motor interference, and *d*: Cohen's *d*; significant effects are displayed in bold.
